# 5-Chloro-5′′-(4-chloro­benzyl­idene)-4′-(4-chloro­phen­yl)-1′,1′′-dimethyldi­spiro­[indoline-3,2′-pyrrolidine-3′,3′′-piperidine]-2,4′′-dione

**DOI:** 10.1107/S1600536814004309

**Published:** 2014-02-28

**Authors:** I. S. Ahmed Farag, Adel S. Girgis, A. A. Ramadan, A. M. Moustafa, Ahmed F. Mabied

**Affiliations:** a Crystallography Laboratory, Solid State Department, Physics Division, National Research Centre, Dokki, Giza 12622, Egypt; b Pesticide Chemistry Department, National Research Centre, Dokki, Giza 12622, Egypt; c Physics Department, Faculty of Science, Helwan University, Helwan, Cairo, Egypt

## Abstract

The racemic title compound, C_30_H_26_Cl_3_N_3_O_2_, comprises two spiro links, the first connecting the piperidine and pyrrolidine rings and the other connecting the indole and pyrrolidine rings. The piperidine ring adopts a half-chair conformation, while the pyrrolidine ring has an envelope conformation with the unsubstituted C atom as the flap. The dihedral angles between the two *p*-Cl-substituted benzene rings and the indole ring are 33.13 (14) and 54.11 (14)°. In the crystal, mol­ecules form inversion dimers through pairs of N—H⋯O hydrogen bonds [graph set *R*_2_^2^(8)]. Aromatic C—H⋯O hydrogen bonds extend these dimers into a ribbon structure, enclosing *R* ^2^_2_(14) ring motifs, along the *a*-axis direction.

## Related literature   

For the biological activity of related di­spiro-oxindole analogues, see: Girgis *et al.* (2009*a* ▶,*b* ▶, 2012 ▶); George *et al.* (2013 ▶). For related structural studies, see: Farag *et al.* (2014*a* ▶,*b* ▶,*c* ▶); Moustafa *et al.* (2012 ▶). For the synthesis of the precursor mol­ecule, see: Modzelewska *et al.* (2006 ▶). For graph-set analysis, see: Etter *et al.* (1990 ▶). For details of the weighting scheme used, see: Watkin *et al.* (1994 ▶). H atoms were refined with riding constraints (Cooper *et al.*, 2010 ▶).
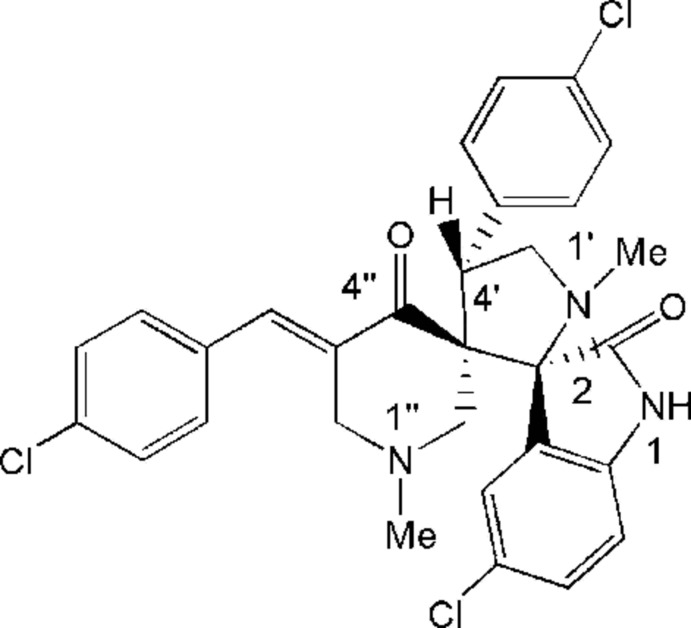


## Experimental   

### 

#### Crystal data  

C_30_H_26_Cl_3_N_3_O_2_
*M* *_r_* = 566.91
Triclinic, 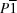

*a* = 11.2102 (3) Å
*b* = 11.5909 (3) Å
*c* = 12.3569 (4) Å
α = 99.0734 (8)°
β = 90.1887 (9)°
γ = 116.4041 (10)°
*V* = 1415.22 (7) Å^3^
*Z* = 2
Mo *K*α radiation
μ = 0.36 mm^−1^
*T* = 298 K
0.35 × 0.19 × 0.10 mm

#### Data collection  

Nonius KappaCCD diffractometer
Absorption correction: multi-scan [Görbitz (1999 ▶) and *DENZO*/*SCALEPACK* (Otwinowski & Minor, 1997 ▶)] *T*_min_ = 0.630, *T*_max_ = 0.876
16419 measured reflections
6508 independent reflections
3663 reflections with *I* > 2σ(*I*)
*R*_int_ = 0.076

#### Refinement  

*R*[*F* ^2^ > 2σ(*F* ^2^)] = 0.057
*wR*(*F* ^2^) = 0.111
*S* = 1.01
3663 reflections
344 parameters
H-atom parameters constrained
Δρ_max_ = 0.55 e Å^−3^
Δρ_min_ = −0.54 e Å^−3^

### 

Data collection: *COLLECT* (Nonius, 2001 ▶).; cell refinement: *DENZO*/*SCALEPACK* (Otwinowski & Minor, 1997 ▶); data reduction: *DENZO*/*SCALEPACK*; program(s) used to solve structure: *SUPERFLIP* (Palatinus & Chapuis, 2007 ▶); program(s) used to refine structure: *CRYSTALS* (Betteridge *et al.*, 2003 ▶); molecular graphics: *CAMERON* (Watkin *et al.*, 1996 ▶) and *DIAMOND* (Brandenburg, 2012 ▶); software used to prepare material for publication: *CRYSTALS*; software used to prepare material for publication: *publCIF* (Westrip, 2010 ▶).

## Supplementary Material

Crystal structure: contains datablock(s) New_Global_Publ_Block, I. DOI: 10.1107/S1600536814004309/zs2286sup1.cif

Structure factors: contains datablock(s) I. DOI: 10.1107/S1600536814004309/zs2286Isup2.hkl

CCDC reference: 988672

Additional supporting information:  crystallographic information; 3D view; checkCIF report

## Figures and Tables

**Table 1:** Hydrogen-bond geometry (Å, °)

*D*—H⋯*A*	*D*—H	H⋯*A*	*D*⋯*A*	*D*—H⋯*A*
C6—H61⋯O19^i^	0.96	2.47	3.168 (5)	130
N30—H301⋯O29^ii^	0.96	1.90	2.844 (5)	167

Symmetry codes: (i) 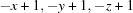; (ii) 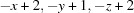.

**Fig. 1.**
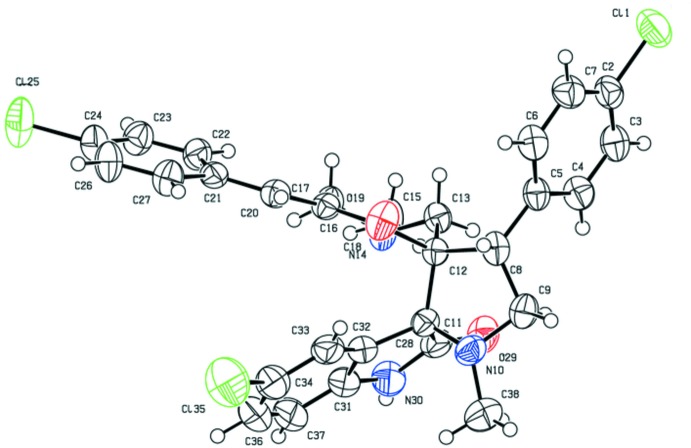
The title compound with displacement ellipsoids drawn at the 50% probability level. H atoms are shown as spheres of arbitrary radius.

**Fig. 2.**
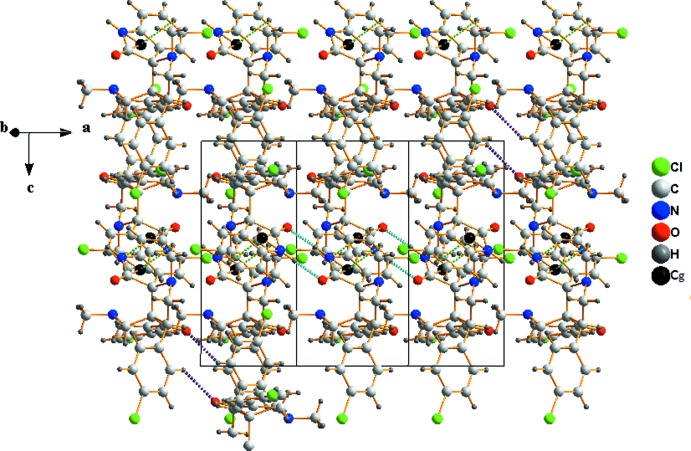
A view of the crystal packing of the title compound, showing the N—H···O and C—H···O hydrogen bonds and the C—H..π interaction as violet, turquoise and green dashed lines, respectively.

## supplementary crystallographic information

### 1. Introduction

Spiro­pyrrolidinyl-oxindole represents the main alkaloid skeleton of naturally occurring substances characterized by promising biological and/or pharmacological properties. In continuation of our research program directed towards synthesis of biologically active compounds possessing this motif (Farag *et al.*, 2014**a*-c*; George *et al.*, 2013; Girgis *et al.*, 2012, 2009**a*,b*; Moustafa *et al.*, 2012), a novel analog, C_30_H_26_Cl_3_N_3_O_2_, is described in the present study utilizing a facile regio- as well as stereoselective procedure.

### 2.1. Synthesis and crystallization

A mixture of equimolar amounts of 3*E*,5*E*-3,5-bis­(4-chloro­phenyl­methyl­idene)-1-methyl-4-piperidone (5 mmol) [prepared by a literature procedure (Modzelewska *et al.*, 2006)], 5-chloro­isatin and sarcosine in absolute ethanol (25 ml) was heated under reflux for 9 h (TLC monitoring). The separated solid was collected and recrystallized from *n*-butanol affording the title compound, 5-chloro-5''-(4-chloro­benzyl­idene)-4'-(4-chloro­phenyl)-1',1''-di­methyl­dispiro­[indoline-3,2'-pyrrolidine-3',3''-piperidine]-2,4''-dione, as pale-yellow crystals. M.p. 237-239 °C; Yield 81%; Anal. Calcd. for C_30_H_26_Cl_3_N_3_O_2_ (566.92): C, 63.56; H, 4.62; N, 7.41. Found: C, 63.69; H, 4.71; N, 7.48. IR: *ν*_max_/cm^-1^ 3164 (NH), 1690 (C═O), 1594, 1483 (C═C).

### 2.2. Refinement

Crystal data, data collection and structure refinement details are summarized in Table 1. The relatively large ratio of minimum to maximum corrections applied in the multiscan process reflect changes in the illuminated volume of the crystal. Changes in illuminated volume were kept to a minimum, and were taken into account (Görbitz, 1999) by the multi-scan inter-frame scaling [DENZO/SCALEPACK (Otwinowski & Minor, 1997)]. The H atoms were all located in a difference map, but those attached to carbon atoms were repositioned geometrically and initially refined with soft restraints on the bond lengths and angles to regularise their geometry (C—H in the range 0.93–0.98, N—H in the range 0.86–0.89, N—H to 0.86 and O—H = 0.82 Å) and *U>*_iso_(H) (in the range 1.2–1.5 times *U*_eq_ of the parent atom), after which the positions were refined with riding constraints (Cooper *et al.*, 2010).

### 3. (Results and Discussion)

In the racemic title molecule (Fig. 1), two spiro links exist, one in which the piperidine and pyrrolidine rings are connected at C12, the other with the pyrrolidine ring and indole residue connected at C11. The piperidine ring adopts a half-chair conformation where the C13 atom lies 0.75 (2) Å out of the mean plane of the remaining five atoms (C12–N14) with maximum deviation 0.086 (2) at C16. The pyrrolidine ring has an envelope conformation with the flap atom being C9 which lies 0.608 (3) Å out of the mean plane of the remaining four atoms (C8–N10), in which the maximum deviation [0.088 (3)] is at C11. The two 4-chloro-substituted benzene rings defined by (C2–C7) and (C21–C27) make dihedral angles of 33.1 (14) and 54.11 (14)°, respectively, with the indole ring. In the crystal, the molecules form centrosymmetric cyclic dimers through duplex inter­molecular N30—H···O29^i^ hydrogen bonds (Table 1) [graph set *R*^2^_2_(8) (Etter *et al.*, 1990)]. Centrosymmetric duplex aromatic C6—H···O19^ii^ hydrogen-bonding associations [graph set *R*^2^_2_(14)] extend these dimers into a one-dimensional ribbon structure extending along *a* (Fig. 2) (for symmetry codes, see Table 1). Also present in the molecule is an intra­molecular C38—H···π inter­action with the ring centroid (*Cg*) of the five-membered C28–N30 ring.

### Crystal data

**Table:**

C_30_H_26_Cl_3_N_3_O_2_	*Z* = 2
*M**_r_* = 566.91	*F*(000) = 588
Triclinic, *P*1	*D*_x_ = 1.330 Mg m^−^^3^
Hall symbol: -P 1	Melting point = 510–512 K
*a* = 11.2102 (3) Å	Mo *K*α radiation, λ = 0.71073 Å
*b* = 11.5909 (3) Å	Cell parameters from 8506 reflections
*c* = 12.3569 (4) Å	θ = 3–27°
α = 99.0734 (8)°	µ = 0.36 mm^−^^1^
β = 90.1887 (9)°	*T* = 298 K
γ = 116.4041 (10)°	Plate, pale yellow
*V* = 1415.22 (7) Å^3^	0.35 × 0.19 × 0.10 mm

### Data collection

**Table:**

Nonius KappaCCD diffractometer	3663 reflections with *I* > 2σ(*I*)
Graphite monochromator	*R*_int_ = 0.076
φ and ω scans	θ_max_ = 27.5°, θ_min_ = 3.4°
Absorption correction: multi-scan [Görbitz (1999) and *DENZO*/*SCALEPACK* (Otwinowski & Minor, 1997)]	*h* = −14→13
*T*_min_ = 0.630, *T*_max_ = 0.876	*k* = −14→15
16419 measured reflections	*l* = −13→16
6508 independent reflections	

### Refinement

**Table:**

Refinement on *F*^2^	Hydrogen site location: difference Fourier map
Least-squares matrix: full	H-atom parameters constrained
*R*[*F*^2^ > 2σ(*F*^2^)] = 0.057	Method, part 1, Chebychev polynomial, (Watkin et al., 1994) [*w*eight] = 1.0/[A_0_*T_0_(x) + A_1_*T_1_(x) ··· + A_n-1_]*T_n-1_(x)] where A_i_ are the Chebychev coefficients listed belo*w* and x = *F* /*F*max Method = Robust Weighting W = [*w*eight] * [1-(delta*F*/6*sigma*F*)^2^]^2^ A_i_ are: 100. 168. 111. 49.9 14.3
*wR*(*F*^2^) = 0.111	(Δ/σ)_max_ = 0.001
*S* = 1.01	Δρ_max_ = 0.55 e Å^−^^3^
3663 reflections	Δρ_min_ = −0.54 e Å^−^^3^
344 parameters	Extinction correction: Larson (1970), Equation 22
0 restraints	Extinction coefficient: 400 (70)

### Fractional atomic coordinates and isotropic or equivalent isotropic displacement parameters (Å^2^)

**Table:**

	*x*	*y*	*z*	*U*_iso_*/*U*_eq_	
Cl1	0.60430 (10)	0.03618 (8)	0.26749 (8)	0.0754	
C2	0.6044 (3)	0.1352 (3)	0.3894 (3)	0.0503	
C3	0.6541 (3)	0.1232 (3)	0.4866 (3)	0.0529	
C4	0.6557 (3)	0.2027 (3)	0.5831 (3)	0.0487	
C5	0.6067 (3)	0.2943 (2)	0.5838 (2)	0.0417	
C6	0.5543 (3)	0.3014 (3)	0.4848 (2)	0.0492	
C7	0.5530 (3)	0.2237 (3)	0.3873 (3)	0.0542	
C8	0.6072 (2)	0.3840 (2)	0.6877 (2)	0.0409	
C9	0.5750 (3)	0.3253 (3)	0.7914 (3)	0.0516	
N10	0.6166 (2)	0.4411 (2)	0.8759 (2)	0.0492	
C11	0.7498 (3)	0.5363 (2)	0.8520 (2)	0.0397	
C12	0.7408 (2)	0.5121 (2)	0.7215 (2)	0.0354	
C13	0.8657 (2)	0.5050 (2)	0.6782 (2)	0.0400	
N14	0.9809 (2)	0.6228 (2)	0.73096 (19)	0.0418	
C15	1.1053 (3)	0.6102 (3)	0.7277 (3)	0.0639	
C16	0.9952 (3)	0.7356 (2)	0.6850 (2)	0.0432	
C17	0.8667 (3)	0.7462 (2)	0.6750 (2)	0.0367	
C18	0.7363 (3)	0.6297 (2)	0.6818 (2)	0.0374	
O19	0.63054 (19)	0.62778 (19)	0.65825 (18)	0.0539	
C20	0.8581 (3)	0.8543 (2)	0.6587 (2)	0.0402	
C21	0.9613 (3)	0.9854 (3)	0.6496 (2)	0.0406	
C22	1.0951 (3)	1.0193 (3)	0.6338 (2)	0.0477	
C23	1.1861 (3)	1.1452 (3)	0.6272 (3)	0.0532	
C24	1.1446 (3)	1.2413 (3)	0.6355 (3)	0.0522	
Cl25	1.25982 (10)	1.40017 (8)	0.62645 (10)	0.0835	
C26	1.0126 (3)	1.2121 (3)	0.6494 (3)	0.0598	
C27	0.9234 (3)	1.0851 (3)	0.6562 (3)	0.0521	
C28	0.8616 (3)	0.5122 (3)	0.9037 (2)	0.0482	
O29	0.8737 (2)	0.4106 (2)	0.88320 (18)	0.0582	
N30	0.9342 (3)	0.6146 (2)	0.9844 (2)	0.0591	
C31	0.8905 (3)	0.7109 (3)	0.9914 (3)	0.0578	
C32	0.7834 (3)	0.6724 (3)	0.9147 (2)	0.0466	
C33	0.7187 (3)	0.7487 (3)	0.9109 (3)	0.0559	
C34	0.7670 (5)	0.8668 (3)	0.9847 (3)	0.0756	
Cl35	0.68788 (17)	0.96581 (12)	0.98031 (12)	0.1253	
C36	0.8741 (5)	0.9054 (4)	1.0601 (3)	0.0925	
C37	0.9380 (5)	0.8278 (4)	1.0642 (3)	0.0812	
C38	0.6049 (4)	0.4161 (4)	0.9884 (3)	0.0741	
H31	0.6875	0.0600	0.4875	0.0634*	
H41	0.6912	0.1947	0.6508	0.0584*	
H61	0.5179	0.3622	0.4839	0.0591*	
H71	0.5171	0.2309	0.3194	0.0651*	
H81	0.5398	0.4108	0.6738	0.0490*	
H91	0.6249	0.2780	0.8011	0.0619*	
H92	0.4813	0.2684	0.7906	0.0619*	
H131	0.8702	0.4292	0.6959	0.0480*	
H132	0.8631	0.5003	0.5999	0.0480*	
H151	1.1779	0.6908	0.7637	0.0769*	
H152	1.0970	0.5405	0.7648	0.0769*	
H153	1.1224	0.5912	0.6526	0.0769*	
H161	1.0599	0.8133	0.7317	0.0518*	
H162	1.0265	0.7293	0.6131	0.0518*	
H201	0.7683	0.8438	0.6517	0.0482*	
H221	1.1247	0.9526	0.6274	0.0572*	
H231	1.2778	1.1659	0.6168	0.0638*	
H261	0.9835	1.2790	0.6542	0.0717*	
H271	0.8317	1.0649	0.6659	0.0624*	
H331	0.6431	0.7215	0.8593	0.0669*	
H361	0.9045	0.9868	1.1105	0.1110*	
H371	1.0133	0.8547	1.1160	0.0974*	
H381	0.6343	0.4974	1.0388	0.0890*	
H382	0.6594	0.3746	1.0027	0.0890*	
H383	0.5134	0.3597	0.9977	0.0890*	
H301	1.0063	0.6204	1.0307	0.0710*	

### Atomic displacement parameters (Å^2^)

**Table:**

	*U*^11^	*U*^22^	*U*^33^	*U*^12^	*U*^13^	*U*^23^
Cl1	0.0825 (6)	0.0603 (5)	0.0685 (6)	0.0259 (4)	0.0069 (5)	−0.0100 (4)
C2	0.0439 (16)	0.0407 (15)	0.0535 (19)	0.0097 (13)	0.0027 (14)	0.0021 (13)
C3	0.0552 (19)	0.0391 (15)	0.067 (2)	0.0250 (14)	0.0025 (16)	0.0058 (14)
C4	0.0498 (17)	0.0480 (16)	0.0542 (18)	0.0276 (14)	−0.0035 (14)	0.0085 (14)
C5	0.0327 (14)	0.0361 (13)	0.0516 (17)	0.0129 (11)	−0.0049 (12)	0.0032 (12)
C6	0.0486 (17)	0.0442 (15)	0.0554 (19)	0.0226 (13)	−0.0095 (14)	0.0053 (14)
C7	0.0500 (18)	0.0528 (17)	0.056 (2)	0.0208 (15)	−0.0087 (14)	0.0062 (15)
C8	0.0321 (14)	0.0375 (13)	0.0520 (17)	0.0160 (11)	−0.0036 (12)	0.0044 (12)
C9	0.0406 (16)	0.0400 (15)	0.065 (2)	0.0101 (13)	0.0063 (14)	0.0100 (14)
N10	0.0506 (14)	0.0472 (13)	0.0492 (15)	0.0193 (11)	0.0139 (11)	0.0150 (11)
C11	0.0409 (15)	0.0392 (13)	0.0391 (16)	0.0179 (12)	0.0033 (12)	0.0083 (11)
C12	0.0338 (14)	0.0341 (12)	0.0392 (15)	0.0161 (11)	−0.0021 (11)	0.0067 (11)
C13	0.0385 (15)	0.0359 (13)	0.0483 (17)	0.0200 (12)	−0.0008 (12)	0.0055 (12)
N14	0.0338 (12)	0.0379 (11)	0.0572 (15)	0.0182 (10)	0.0009 (10)	0.0117 (10)
C15	0.0433 (17)	0.0531 (18)	0.105 (3)	0.0271 (15)	0.0038 (18)	0.0238 (19)
C16	0.0409 (15)	0.0358 (13)	0.0547 (18)	0.0187 (12)	0.0050 (13)	0.0090 (12)
C17	0.0420 (14)	0.0401 (14)	0.0332 (14)	0.0227 (12)	0.0008 (11)	0.0072 (11)
C18	0.0391 (15)	0.0421 (14)	0.0345 (14)	0.0222 (12)	−0.0022 (11)	0.0043 (11)
O19	0.0432 (12)	0.0533 (12)	0.0723 (15)	0.0251 (10)	−0.0040 (10)	0.0206 (10)
C20	0.0426 (15)	0.0426 (14)	0.0399 (16)	0.0227 (12)	0.0008 (12)	0.0091 (12)
C21	0.0491 (17)	0.0409 (14)	0.0338 (15)	0.0222 (13)	−0.0027 (12)	0.0059 (11)
C22	0.0560 (18)	0.0423 (15)	0.0528 (18)	0.0289 (14)	0.0095 (14)	0.0096 (13)
C23	0.0528 (18)	0.0480 (16)	0.064 (2)	0.0260 (15)	0.0117 (15)	0.0129 (14)
C24	0.0551 (19)	0.0389 (14)	0.060 (2)	0.0192 (13)	0.0050 (15)	0.0075 (13)
Cl25	0.0753 (6)	0.0397 (4)	0.1315 (9)	0.0217 (4)	0.0239 (6)	0.0166 (5)
C26	0.066 (2)	0.0391 (15)	0.080 (2)	0.0298 (15)	0.0029 (17)	0.0089 (15)
C27	0.0483 (17)	0.0483 (16)	0.066 (2)	0.0277 (14)	−0.0018 (14)	0.0101 (14)
C28	0.0530 (18)	0.0491 (16)	0.0441 (17)	0.0219 (14)	0.0003 (13)	0.0164 (14)
O29	0.0642 (14)	0.0510 (12)	0.0678 (14)	0.0315 (11)	−0.0091 (11)	0.0171 (10)
N30	0.0666 (17)	0.0545 (15)	0.0520 (16)	0.0234 (13)	−0.0198 (13)	0.0101 (13)
C31	0.074 (2)	0.0489 (17)	0.0404 (17)	0.0196 (16)	−0.0052 (15)	0.0071 (14)
C32	0.0567 (17)	0.0461 (15)	0.0370 (16)	0.0218 (14)	0.0102 (13)	0.0110 (13)
C33	0.074 (2)	0.0544 (17)	0.0493 (19)	0.0361 (16)	0.0206 (16)	0.0134 (14)
C34	0.115 (3)	0.059 (2)	0.066 (2)	0.050 (2)	0.029 (2)	0.0090 (19)
Cl35	0.1911 (15)	0.0969 (8)	0.1324 (11)	0.1058 (10)	0.0520 (10)	0.0150 (8)
C36	0.153 (4)	0.057 (2)	0.054 (2)	0.040 (3)	0.007 (3)	−0.0053 (18)
C37	0.119 (3)	0.061 (2)	0.043 (2)	0.025 (2)	−0.011 (2)	−0.0012 (17)
C38	0.085 (3)	0.074 (2)	0.062 (2)	0.030 (2)	0.0281 (19)	0.0254 (19)

### Geometric parameters (Å, º)

**Table:**

Cl1—C2	1.743 (3)	C16—H162	0.960
C2—C3	1.375 (4)	C17—C18	1.500 (4)
C2—C7	1.386 (4)	C17—C20	1.345 (3)
C3—C4	1.382 (4)	C18—O19	1.209 (3)
C3—H31	0.960	C20—C21	1.467 (4)
C4—C5	1.394 (4)	C20—H201	0.960
C4—H41	0.960	C21—C22	1.395 (4)
C5—C6	1.386 (4)	C21—C27	1.389 (4)
C5—C8	1.517 (4)	C22—C23	1.376 (4)
C6—C7	1.382 (4)	C22—H221	0.960
C6—H61	0.960	C23—C24	1.375 (4)
C7—H71	0.960	C23—H231	0.960
C8—C9	1.514 (4)	C24—Cl25	1.739 (3)
C8—C12	1.563 (3)	C24—C26	1.381 (4)
C8—H81	0.960	C26—C27	1.380 (4)
C9—N10	1.452 (4)	C26—H261	0.960
C9—H91	0.960	C27—H271	0.960
C9—H92	0.960	C28—O29	1.230 (3)
N10—C11	1.474 (3)	C28—N30	1.352 (4)
N10—C38	1.459 (4)	N30—C31	1.397 (4)
C11—C12	1.588 (4)	N30—H301	0.960
C11—C28	1.556 (4)	C31—C32	1.385 (4)
C11—C32	1.521 (4)	C31—C37	1.378 (5)
C12—C13	1.532 (4)	C32—C33	1.376 (4)
C12—C18	1.540 (3)	C33—C34	1.393 (5)
C13—N14	1.450 (3)	C33—H331	0.960
C13—H131	0.960	C34—Cl35	1.741 (4)
C13—H132	0.960	C34—C36	1.376 (6)
N14—C15	1.464 (3)	C36—C37	1.382 (6)
N14—C16	1.452 (3)	C36—H361	0.960
C15—H151	0.960	C37—H371	0.960
C15—H152	0.960	C38—H381	0.960
C15—H153	0.960	C38—H382	0.960
C16—C17	1.505 (4)	C38—H383	0.960
C16—H161	0.960		
			
Cl1—C2—C3	119.5 (2)	C17—C16—H161	108.5
Cl1—C2—C7	119.7 (3)	N14—C16—H162	108.5
C3—C2—C7	120.8 (3)	C17—C16—H162	108.5
C2—C3—C4	119.5 (3)	H161—C16—H162	109.5
C2—C3—H31	120.2	C16—C17—C18	119.6 (2)
C4—C3—H31	120.2	C16—C17—C20	124.7 (2)
C3—C4—C5	121.2 (3)	C18—C17—C20	115.6 (2)
C3—C4—H41	119.4	C12—C18—C17	117.8 (2)
C5—C4—H41	119.4	C12—C18—O19	120.7 (2)
C4—C5—C6	117.8 (3)	C17—C18—O19	121.5 (2)
C4—C5—C8	122.9 (3)	C17—C20—C21	131.5 (3)
C6—C5—C8	119.3 (2)	C17—C20—H201	114.2
C5—C6—C7	121.9 (3)	C21—C20—H201	114.3
C5—C6—H61	119.0	C20—C21—C22	125.7 (2)
C7—C6—H61	119.0	C20—C21—C27	117.8 (3)
C2—C7—C6	118.7 (3)	C22—C21—C27	116.5 (2)
C2—C7—H71	120.6	C21—C22—C23	122.1 (3)
C6—C7—H71	120.7	C21—C22—H221	119.0
C5—C8—C9	116.7 (2)	C23—C22—H221	119.0
C5—C8—C12	115.4 (2)	C22—C23—C24	119.4 (3)
C9—C8—C12	104.3 (2)	C22—C23—H231	120.3
C5—C8—H81	106.6	C24—C23—H231	120.3
C9—C8—H81	106.6	C23—C24—Cl25	119.3 (2)
C12—C8—H81	106.6	C23—C24—C26	120.7 (3)
C8—C9—N10	101.9 (2)	Cl25—C24—C26	120.1 (2)
C8—C9—H91	111.3	C24—C26—C27	118.8 (3)
N10—C9—H91	111.3	C24—C26—H261	120.6
C8—C9—H92	111.3	C27—C26—H261	120.6
N10—C9—H92	111.3	C21—C27—C26	122.5 (3)
H91—C9—H92	109.5	C21—C27—H271	118.7
C9—N10—C11	107.0 (2)	C26—C27—H271	118.7
C9—N10—C38	114.9 (2)	C11—C28—O29	125.7 (3)
C11—N10—C38	115.6 (3)	C11—C28—N30	108.9 (2)
N10—C11—C12	103.0 (2)	O29—C28—N30	125.0 (3)
N10—C11—C28	110.9 (2)	C28—N30—C31	111.0 (2)
C12—C11—C28	113.1 (2)	C28—N30—H301	124.5
N10—C11—C32	110.3 (2)	C31—N30—H301	124.5
C12—C11—C32	119.1 (2)	N30—C31—C32	110.5 (3)
C28—C11—C32	100.6 (2)	N30—C31—C37	128.0 (3)
C8—C12—C11	103.6 (2)	C32—C31—C37	121.5 (3)
C8—C12—C13	115.0 (2)	C11—C32—C31	108.8 (2)
C11—C12—C13	111.2 (2)	C11—C32—C33	130.2 (3)
C8—C12—C18	111.9 (2)	C31—C32—C33	120.7 (3)
C11—C12—C18	108.90 (19)	C32—C33—C34	117.6 (3)
C13—C12—C18	106.2 (2)	C32—C33—H331	121.2
C12—C13—N14	107.5 (2)	C34—C33—H331	121.2
C12—C13—H131	109.9	C33—C34—Cl35	118.5 (4)
N14—C13—H131	109.9	C33—C34—C36	121.5 (3)
C12—C13—H132	110.0	Cl35—C34—C36	120.0 (3)
N14—C13—H132	110.0	C34—C36—C37	120.6 (3)
H131—C13—H132	109.5	C34—C36—H361	119.7
C13—N14—C15	113.2 (2)	C37—C36—H361	119.7
C13—N14—C16	111.3 (2)	C36—C37—C31	118.1 (4)
C15—N14—C16	110.6 (2)	C36—C37—H371	121.0
N14—C15—H151	109.5	C31—C37—H371	121.0
N14—C15—H152	109.5	N10—C38—H381	109.5
H151—C15—H152	109.5	N10—C38—H382	109.5
N14—C15—H153	109.4	H381—C38—H382	109.5
H151—C15—H153	109.5	N10—C38—H383	109.4
H152—C15—H153	109.5	H381—C38—H383	109.5
N14—C16—C17	113.3 (2)	H382—C38—H383	109.5
N14—C16—H161	108.5		
			
Cl1—C2—C7—C6	−179.9 (3)	C11—C32—C33—C34	175.1 (3)
C34—C36—C37—C31	−0.1 (6)	C20—C21—C27—C26	179.6 (3)
C12—C13—N14—C15	160.2 (2)	O29—C28—N30—C31	−175.9 (3)
C8—C12—C18—O19	20.1 (3)	N30—C31—C32—C11	1.4 (4)
N14—C16—C17—C18	−16.4 (3)		

### Hydrogen-bond geometry (Å, º)

**Table:**

*D*—H···*A*	*D*—H	H···*A*	*D*···*A*	*D*—H···*A*
C6—H61···O19^i^	0.96	2.47	3.168 (5)	130
N30—H301···O29^ii^	0.96	1.90	2.844 (5)	167
C38—H381···*Cg*	0.95	2.63	2.818 (5)	91 (1)

Symmetry codes: (i) −*x*+1, −*y*+1, −*z*+1; (ii) −*x*+2, −*y*+1, −*z*+2.
